# Three spectrophotometric quantitative analysis of bisoprolol fumarate and telmisartan in fixed-dose combination utilizing ratio spectra manipulation methods

**DOI:** 10.1038/s41598-024-72525-6

**Published:** 2024-10-02

**Authors:** Osama I. Abdel Sattar, Hamed H. M. Abuseada, Sherif Ramzy, Mahmoud M. Abuelwafa

**Affiliations:** Pharmaceutical Analytical Chemistry Department, Faculty of Pharmacy, Al-Azhar University, Nasr City, 11751 Cairo Egypt

**Keywords:** Bisoprolol fumarate, First derivative of ratio spectra, Mean centering of ratio spectra, Ratio difference, Telmisartan, UV spectrophotometry, Analytical chemistry, Green chemistry

## Abstract

Hypertension is a chronic condition with multiple drug regimens. Limiting these medicines is critical to patient compliance. Therefore, bisoprolol and telmisartan were recently developed in a fixed-dose combination to control blood pressure. The UV absorption spectra of bisoprolol and telmisartan overlapped significantly. Thus, three spectrophotometric methods have been developed for simultaneous determination of bisoprolol and telmisartan without prior separation. Method A is ratio difference of ratio spectra (RD), which measures the amplitude difference between (210–224) nm for bisoprolol and between (255–365) nm for telmisartan. Method B, the first derivative of ratio spectra (^1^DD), measures amplitude signals at 232 and 243 nm for bisoprolol and telmisartan, respectively. Method C is the mean centering of ratio spectra (MC), which measures the mean-centered ratio spectra's values at 223 nm for bisoprolol and 245 nm for telmisartan. The applied methods showed good linearity 2–20 µg/mL for bisoprolol, 4–32 µg/mL for telmisartan, with sufficient accuracy and precision. The methods were sensitive, with LOD values of 0.243 µg/mL and 0.596 µg/mL in RD method, 0.313 µg/mL and 0.914 µg/mL in ^1^DD method, and 0.406 and 0.707 µg/mL in MC method for bisoprolol and telmisartan, respectively, the methods were validated per ICH criteria. The novel methods are precise and accurate and can be used for routine analysis and quality control of bisoprolol and telmisartan in pure and dosage form. Furthermore, the greenness of the approaches was evaluated using Analytical Greenness assessment (AGREE), and the suggested method received a high greenness score.

## Introduction

Hypertension is a common cardiovascular risk factor. About 10.4 million people die yearly from high systolic blood pressure (BP)^1^. Despite good medication, many hypertensive patients have poor BP control. Drug nonadherence is a key cause of poor BP control in treated hypertensives. Less than half of the individuals adhere to their antihypertensive medication within one year of starting treatment. The consequences of nonadherence include hypertension-related end-organ damage (microalbuminuria, stroke, and heart failure), higher hospital admissions, lower quality of life, and mortality.The fixed-dose combination therapy (FDC) improves adherence and persistence. They reduce drug adverse effects, improve compliance, and offer sensible combination therapy. These factors lower blood pressure and minimise antihypertensive therapy discontinuations^2^. As a result, bisoprolol fumarate and telmisartan have recently been developed in a fixed-dose combination rather than individual doses to control blood pressure.

**Fig. 1:**
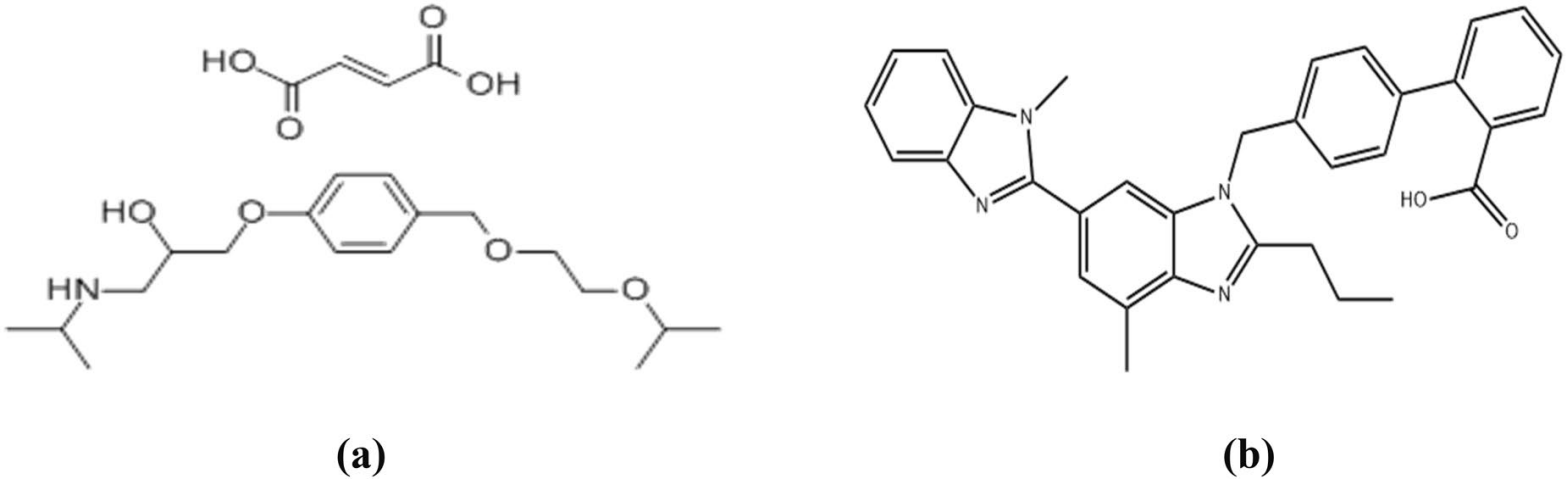
Chemical structures of BPL (**a**), and TST (**b**).

To address this, bisoprolol fumarate (BPL) and telmisartan (TST) have been recently manufactured in a fixed-dose combination to control blood pressure rather than individual doses. Both drugs are antihypertensive agents with distinct modes of action;

bisoprolol fumarate (E)-but-2-enedioic acid;1-(propan-2-ylamino)-3-[4-(2-propan-2-yloxyethoxymethyl)phenoxy]propan-2-ol (Fig. 1a) exerts its effects by specifically and competitively binding to beta-1 adrenergic receptors in the heart. This action leads to a decrease in the heart's contractility and rate, resulting in a reduction in cardiac output and a lowering blood pressure^3,4^. While telmisartan 2-[4-[[4-methyl-6-(1-methylbenzimidazol-2-yl)-2-propylbenzimidazol-1-yl]methyl]phenyl]benzoic acid (Fig. 1b) is an ARB medication prescribed for the treatment of hypertension. Angiotensin II receptor blockers(ARBs), like telmisartan, have a strong affinity for the angiotensin II type 1 (AT1) receptors. This binding inhibits the effects of angiotensin II on the smooth muscles in blood vessels, resulting of a decrease in arterial blood pressure^5–8^.

In addition to improving patient adherence to therapy, fixed-dose combinations are more effective at treating hypertension than monotherapy^1,2,9–11^.

The literature reviews indicate that the established techniques for quantitative determination of BPL and TST in their fixed dose combination are RP-HPLC^12–14^, UV spectrophotometry based on the area under the curve (AUC)^15^, and HP-TLC^8^

The employed three ratio manipulating spectrophotometric methods provide effective analytical tools for determining the composition of this novel mixture as they are simple, straightforword, accurate, don’t need prior separation, and suitable for determination of overlabbed spectra of studied drugs, On ther hand each method has their limits as shown in (Table 1)^16–19^, They are regarded as the optimal choice for regular or quality control analysis of bisoprolol fumarate and telmisartan in their pure form or in pharmaceutical formulation.

**Table 1:** Advantages and limitations of the proposed methods.

Method	Advantages	Limitations
RD	Simple, straightforword Fast, accurate Suitable for determination of multiple components Don’t require prior sparation of components Suitable for determination of overlabbed spectra Provide a large selection of wavelength pairings for selection Enhances the variation in absorbance, facilitating the identification of low quantities of the analyte	Requires cautious selection of suitable wavelengths Sensitivity to noise in date Requires accure selection of divisor
^1^DD	Optimizes resolution by examining derivative signals at certain wavelengths It enables the identification of particular items by analyzing their derivative signals Suitable for detection of ternary mixtures	Requires accurate determination of zero-crossing points
MC	Repair baseline distortions and fluctuations Reduces matrix effects, which are sample components interfering	Requiress accurate baseline correction and accurate determination of mean spectrum

The objective of this publication is to address the issue of spectral overlap and accurately quantify the concentrations of BPL and TST in their pure forms and in their combination. This is achieved by developing three spectrophotometric methods that rely on manipulating ratio spectra, specifically the ratio difference (RD), first derivative (^1^DD) of the ratio spectra, and mean centering (MC) of the ratio spectra.^20–25^

In order to evaluate the environmental friendliness of the methodologies used, the Analytical GREEnness (AGREE)^26,27^ framework has been utilised. The results demonstrate a strong adherence of the applied approach to the principles of Green Analytical Chemistry.

## Experimental

### Materials and solvents

Pure BPL (99.46%) provided by Pharco Pharmaceuticals, Alexandria, Egypt. Pure TST (99.63%) supplied from Hochster Pharmaceutical industries, Badr, Egypt. The tablets were prepared in laboratory, each one containing 5 mg of BPL and 40 TST, 7 mg of magnesium stearate, 15 mg of maize starch, and 20 mg talc powder. We employed double-distilled water and HPLC-analytical-grade methanol as a solvent, which was acquired from Sigma-Aldrich (Darmstadt, Germany). The analysis procedure used reagents and substances of analytical grade.

### Apparatus

For all measurements, a Shimadzu UV–visible 1800 spectrophotometer dual beam (Tokyo, Japan) was employed, and samples' absorption spectra were examined using 10 mm × 3.5 mL quartz cuvettes. The mathematical handling of scanned spectra was carried out for the ratio difference and first derivative of ratio spectra using Shimadzu UV Probe software version 2.1. To implement the mean centering (MC) of ratio spectra using Matlab^®^ R21013b (8.2.0.701) and PLS toolbox software version 2.1.

### Standard stock solutions

Freshly prepared standard stock solutions of BPL and TST containing 100 µg/mL were made by vigorously mixing 10 mg of each drug powder with 50 mL of methanol in a pair of 100-mL volumetric flasks, then adding distilled water to the mark.

### Procedures

#### Linearity and development of calibration graphs

Separate serial dilutions of BPL and TST were made by transferring aliquots of 0.2–2 mL and 0.4–3.2 mL of the respective substances from their standard solutions (100 µg/mL) into two sets of 10 mL volumetric flasks and diluting to volume with double distilled water.

These dilutions' absorption spectra were measured and recorded in the 200–400 nm wavelength range using water as a blank. To develop the ratio spectra, the absorption of the zero-order spectra of the studied drugs was divided by an appropriate divisor spectrum from the second drug's spectra. For BPL ratio spectra, the ideal spectrum was 16 µg/mL TST as a divisor, whereas for TST ratio spectra, the ideal spectrum was 10 µg/mL BPL as a divisor.

##### Ratio difference (RD)

To create the calibration graph and derive the regression equation, the difference in the amplitudes of the acquired ratio spectra at 210 and 224 nm for BPL and at 255 and 265 nm for TST was plotted against the comparable concentrations in µg/mL^24,25^.

##### *First derivative (*^*1*^*DD) of the ratio spectra*

The resulting ratio spectrum for each drug was converted into its first-order derivative using scaling factors 10 and ∆λ = 4. The ^1^DD amplitude values for BPL and TST were recorded at 232 and 243 nm, respectively. The recorded data were plotted against each corresponding drug concentration in µg/mL to produce calibration graphs and the accompanying regression equations^28,29^.

##### Mean centering (MC) of the ratio spectra

With the aid of MATLAB software, the generated ratio spectra in the 200–305 nm range were mean-centered. A wavelength of 223 nm was found for BPL and 245 nm for TST in the mean-centered spectra, respectively. After creating a calibration curve between the mean-centered value and the equivalent concentration in µg/mL, the regression equation was calculated.^30^

#### Application to synthetic mixtures

Five synthetic mixtures of BPL and TST were prepared, and drug concentrations were calculated using quantitative analysis of these mixtures, following the guidelines provided by each method.

#### Application to laboratory-prepared tablets

Concor-T^®^ Tablet, manufactured by Merck Ltd. in India, contains 40 mg of telmisartan and 5 mg of bisoprolol fumarate. These tablets are not available in the local market in Egypt. Therefore, we collaborated with the pharmaceutics department to prepare ten tablets in the laboratory. Each tablet containing 5 mg of BPL and 40 TST, 7 mg of magnesium stearate, 15 mg of maize starch, and 20 mg talc powder.

The ten laboratory-prepared tablets were weighed and coarsely powdered. The weight of a single pill was accurately measured and then moved to a volumetric flask with a capacity of 100 mL. After that, adding 50 mL methanol. Subsequently, a 20-min sonication and filtration process was conducted using Whatman filter paper No. 41, and then the volume was adjusted with distilled water^31^.

In order to obtain a wide range of concentrations for both BPL and TST, further dilutions were prepared using water. The steps detailed in the calibration graph approach for each method were repeated. The regression equation was utilized to ascertain the concentration of each medication.

## Results and discussion

**Fig. 2:**
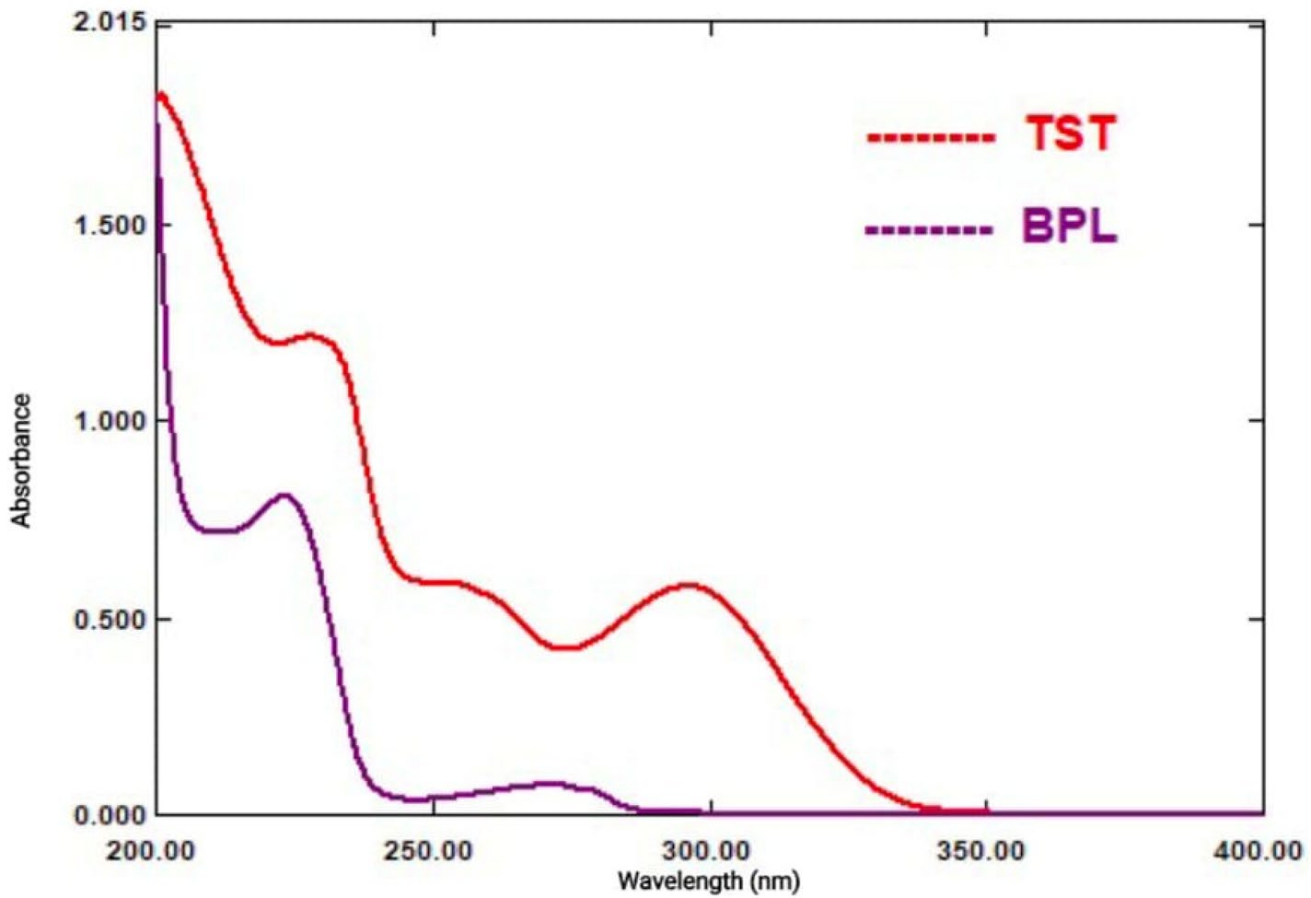
Absorption spectra of BPL (16 μg/mL) and TST (16 μg/mL).

Due to the significant overlap in the UV absorption spectra of BPL and TST, it is quite difficult to determine them simultaneously (Fig. 2). To get beyond the overlap problem and quantitatively identify BPL and TST in their combined mixture, three ratio spectra manipulation techniques were utilized: ratio difference (RD), first derivative (^1^DD) of ratio spectra, and mean centering (MC) of ratio spectra.

These three methods depend on the manipulation of ratio spectra; at the beginning, to generate ratio spectra for each drug, the zero-order spectrum of each drug is divided by the spectrum of another one as a divisor. The selection of the divisor spectrum is a very critical step in minimizing noise in the ratio signal and optimizing sensitivity.

When testing BPL variant concentrations (4, 10, and 16 µg/mL) as divisors, we found that concentration 10 µg/mL was the optimal one. However, when testing different concentrations (12, 16, and 20 µg/mL) of TST as divisors, we discovered that concentration 16 µg/mL was the best one.

### Ratio difference (RD)

**Fig. 3:**
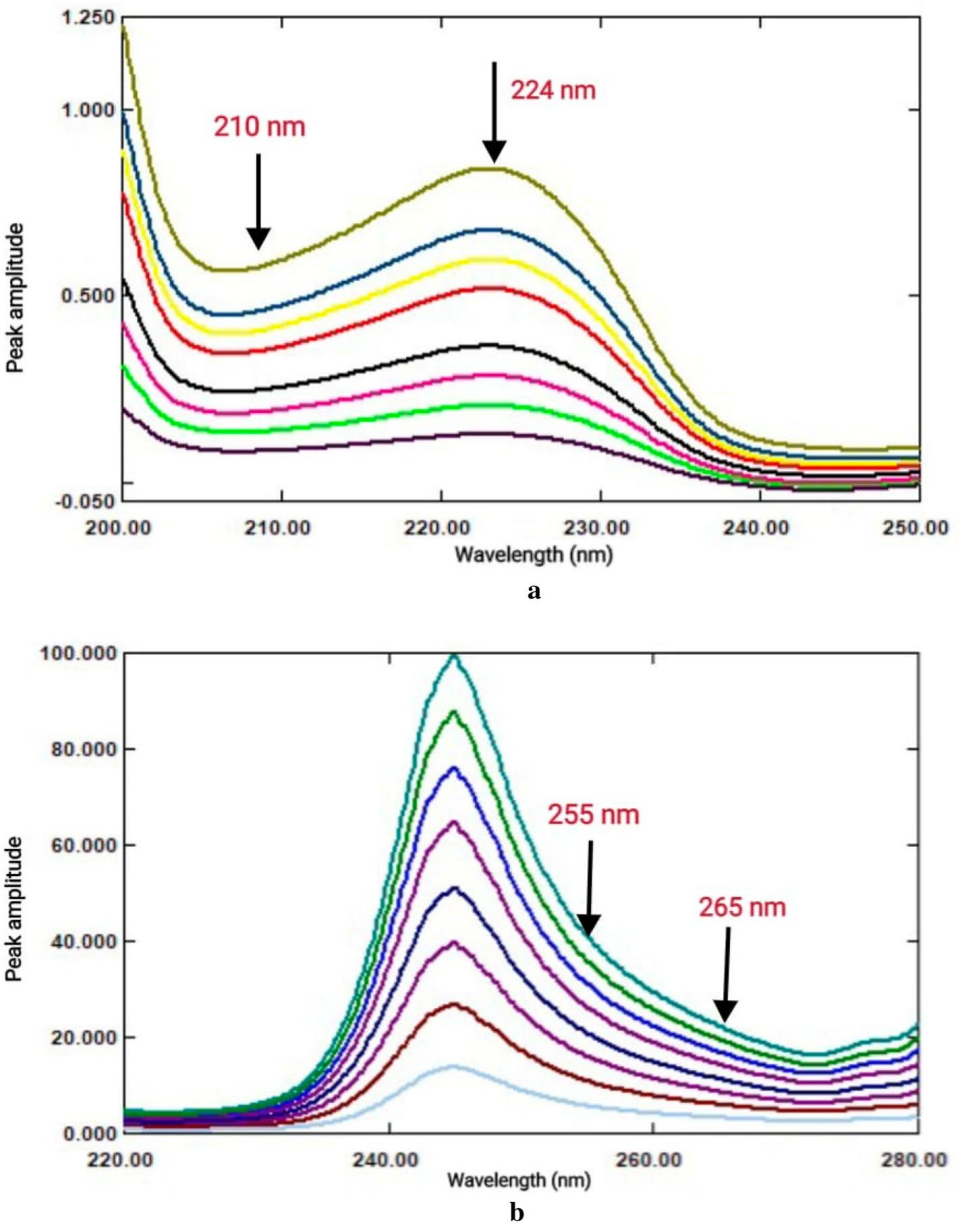
(**a**) Ratio spectra of BPL (2–20 µg/mL) using 16 µg/mL of TST as a divisor. (**b**) Ratio spectra of TST (4–32 µg/mL) using 10 µg/mL of BPL as a divisor.

The ratio difference method: This method depends on computing. The difference in the amplitude values of the ratio spectra at the chosen wavelength pair. Checking the linearity at each chosen wavelength is therefore essential. Following an analysis of the amplitude values' linearity at various wavelengths for the ratio spectra of BPL and TST, it was found that for BPL 210 and 224 nm and for TST 255 and 265 nm produced good linearity with the lowest signal to noise ratio.Thus, BPL could be quantified selectively in a combination by computing the amplitude value differences between 210 and 224 nm without TST interference (Fig. 3a), and TST could be quantified selectively in a combination by computing the amplitude value differences between 255 and 265 nm without TST interference (Fig. 3b).To generate calibration graphs, the amplitude differences calculated for BPL and TST at the chosen wavelengths were graphed against the drug concentrations in µg/mL. Regression equations were then obtained, and the amounts of each drug in the combination were determined.

### *First derivative (*^*1*^*DD) of ratio spectra*

With a scaling factor 10 and ∆λ = 4, the generated ratio spectra of every medication were converted to their first order derivative. Upon analyzing the ^1^DD spectra of BPL and TST at different peak amplitudes, it was shown that the wavelengths of 232 nm and 243 nm, respectively, provided a good combination of linearity and selectivity.

**Fig. 4:**
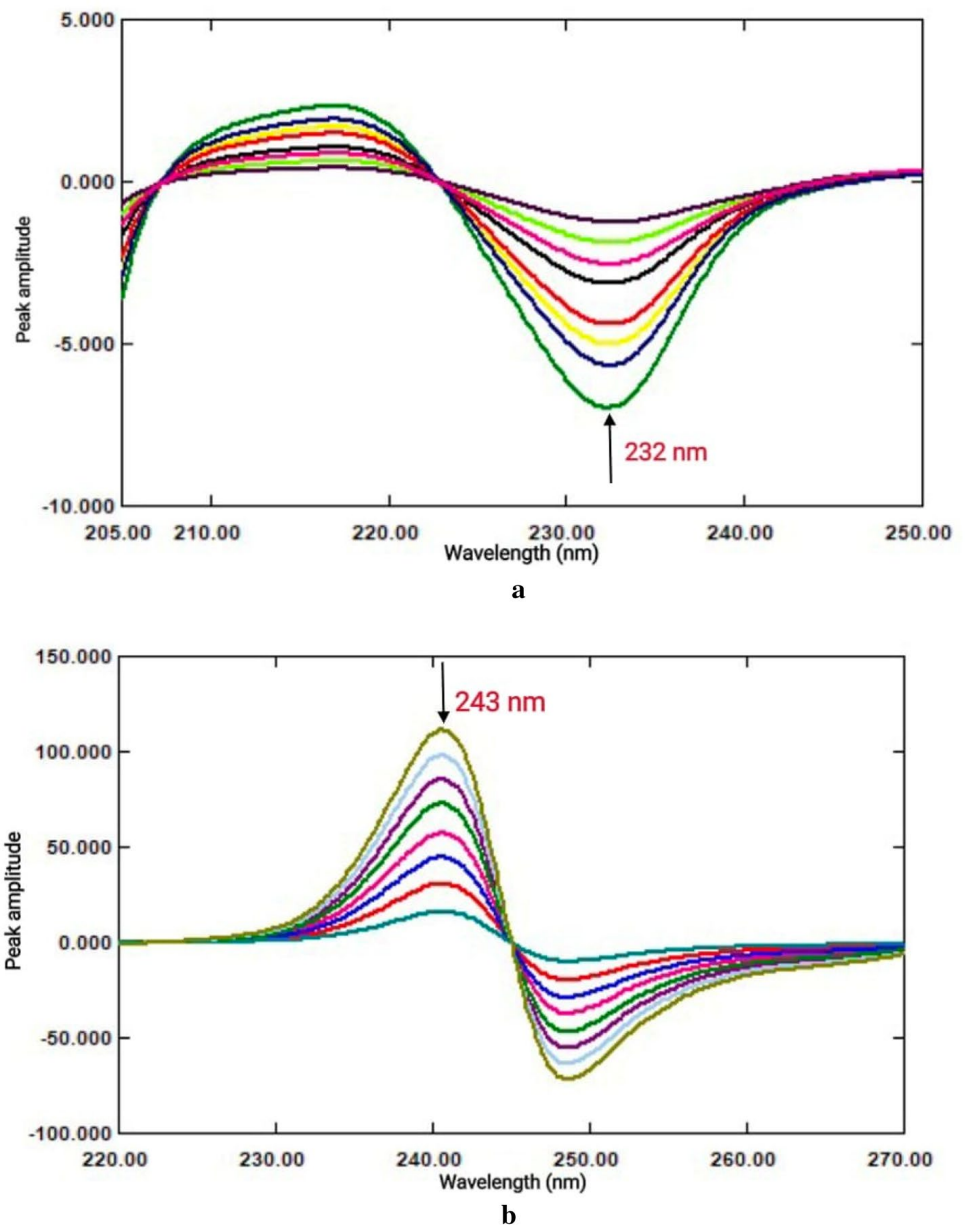
(**a**) ^1^DD spectra of BPL (2–20 µg/mL) using 16 µg/mL of TST as a divisor. (**b**) ^1^DD spectra of TST (4–32 µg/mL) using 10 µg/mL of BPL as a divisor.

Consequently, BPL and TST could be evaluated separately in mixtures at 232 nm and 243 nm, respectively, without disrupting one another (Fig. 4a,b). The calibration graphs were produced by displaying the measured ^1^DD amplitude values of BPL and TST at the chosen wavelengths against the drug concentrations in µg/mL. Regression equations were then obtained, and the amounts of each drug in the combination were determined.

### Mean centering (MC) of the ratio spectra

With MATLAB software, the generated ratio spectra for the studied drugs were mean centered. After analyzing the mean-centered values for BPL and TST, it was shown that wavelengths of 223 nm and 245 nm, respectively, provide good linearity and selectivity.

**Fig. 5:**
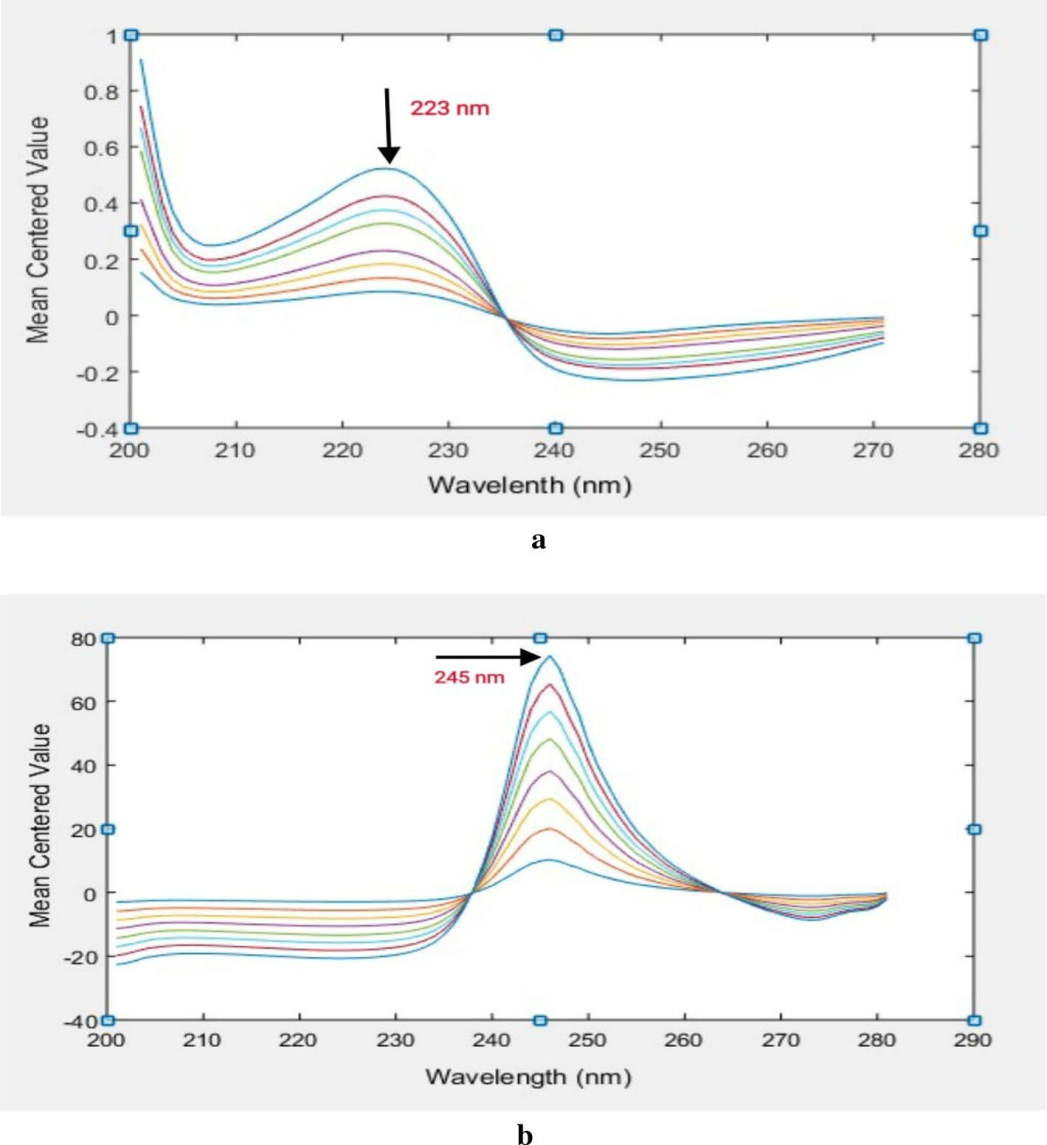
(**a**) Mean centered ratio spectra of BPL (2–20 µg/mL) using 16 µg/mL of TST as a divisor. (**b**) Mean centered ratio spectra of TST (4–32 µg/mL) using 10 µg/mL of BPL as a divisor.

Therefore, BPL and TST could be evaluated separately in mixtures at 223 nm and 245 nm, respectively, without interfering with one another (Fig. 5a,b).

The calibration graphs were created by graphing the mean-centered amplitudes of BPL and TST at the chosen wavelengths against the drug concentrations in µg/mL. Regression equations were then obtained, and the amounts of each drug in the combination were determined.

## Methods of validation

Method validation was performed according to ICH guidelines^32^ for the proposed methods as follows.

### Range and linearity

The linearity of the proposed methods was assessed by utilizing eight concentrations of BPL and TST ranging from 2–20 µg/mL and 4–32 µg/mL, respectively.

In the ratio difference (RD) method, a linear relationship was established for BPL by plotting the amplitude difference between 210 and 224 nm versus the corresponding concentrations, showing 0.9999 as a correlation coefficient, and for TST by graphing the amplitude difference between 255 and 265 nm versus the corresponding concentrations, showing 0.9997 as a correlation coefficient.

In the first derivative (^1^DD) of the ratio spectra method, similarly, by plotting the peak amplitude values at 232 nm and 243 nm for BPL and TST, respectively, versus the respective drug concentrations, with a correlation coefficient of 0.9999 and 0.9998 for BPL and TST, respectively, a linear association was established.

In the mean centering (MC) of the ratio spectra method, likewise, a linear relationship was created by graphing the mean-centered amplitudes of BPL and TST at 223 nm and 245 nm, respectively, against the drug concentrations, with a correlation coefficient of 0.9999 and 0.9995 for BPL and TST, respectively.

A summary of the linear equations was found in Table 1.

### Limits of detection and quantitation

Using the residual standard deviation (σ) and a slope (S), the employed methods' limit of detection (LOD = 3.3σ/S) and limit of quantification (LOQ = 10σ/S) were determined. The computed LOD and LOQ values are shown in Table 2.

**Table 2:** Regression and validation parameters for determination of BPL and TST by the proposed methods.

Parameter	BPL			TST		
	RD	^1^DD	MC	RD	^1^DD	MC
Wavelength (nm)	224–210	232	223	265–255	243	245
Linearity range (µg/mL)	2–20	2–20	2–20	4–32	4–32	4–32
Slope	0.0110	0.0315	0.0243	0.5845	2.2634	2.2829
Intercept	0.0206	0.0617	0.0363	0.2964	1.8242	1.7896
Coefficient of determination (r^2^)	0.9999	0.9999	0.9999	0.9997	0.9998	0.9995
LOD (µg/mL)	0.2433	0.3128	0.4056	0.5965	0.9141	0.7066
LOQ (µg/mL)	0.7372	0.9478	1.2291	1.8076	2.7700	2.1411

### Accuracy

The accuracy of the employed methods was estimated by determining the mean % recoveries of three replicate determinations of five synthetic mixtures (n = 15). The methods produced good accuracy results, as shown in Table 3.

**Table 3:** Accuracy and repeatability precision of the proposed methods in determination of BPL and TST.

Intra-day (within day)							
Conc. of BPL	Conc. of TST	BPL			TST		
		RD	^1^DD	MC	RD	^1^DD	MC
(µg/mL)	(µg/mL)	%R	%R	%R	%R	%R	%R
4	32	100.24	100.19	99.98	99.24	99.47	99.33
3.5	28	99.80	100.15	99.65	98.92	99.93	99.33
3	24	99.94	99.87	99.94	100.65	100.31	99.75
2.5	20	100.39	100.79	100.72	100.13	100.05	99.47
2	16	100.51	100.59	100.78	99.68	100.31	99.69
Accuracy (mean %R)^a^		100.17	100.32	100.21	99.72	100.01	99.51
Repeatability precission (RSD)^b^		0.298	0.366	0.505	0.692	0.346	0.198

^a^Average of 15 determinations (5 concentrations repeated 3 times).

^b^RSD of 15 determinations (5 concentrations repeated 3 times).

### Precision

The precision of the employed methods was determined as the relative standard deviation (RSD) of three replicate determinations of the five synthetic mixtures (n = 15) within a same day for repeatability precision (Table 3) and between three days for intermediate precision (Table 4). The techniques exhibited a high degree of precision, achieving an RSD of under 2%.

**Table 4:** Intermediate precision of the proposed methods in determination of BPL and TST.

Inter-day (between days)							
Conc. of BPL	Conc. of TST	BPL			TST		
		RD	^1^DD	MC	RD	^1^DD	MC
(µg/mL)	(µg/mL)	%R	%R	%R	%R	%R	%R
4	32	100.22	100.04	100.44	100.63	100.30	99.24
3.5	28	99.65	101.18	99.54	99.03	101.31	100.69
3	24	100.56	101.28	101.03	101.10	100.88	100.27
2.5	20	99.96	100.89	100.24	100.80	99.60	98.94
2	16	101.08	100.54	101.33	100.77	101.45	100.81
Mean %R^a^		100.29	100.79	100.51	100.46	100.71	99.99
Intermediate precision (RSD)^b^		0.549	0.503	0.697	0.818	0.759	0.852

^a^Average of 15 determinations (5 concentrations repeated 3 times).

^b^RSD of 15 determinations (5 concentrations repeated 3 times).

### Selectivity

The proposed methods showed good accuracy and precision in addition to great selectivity for the determination of BPL and TST in their synthetic mixed solutions (Table 3) without any influence from each other, and in lab prepared tablets (Table 5). Moreover, Table 5's results for the standard addition technique validate the methods' selectivity.

**Table 5:** Application of the proposed methods for the determination of BPL and TST in laboratory-prepared tablets.

Conc. of BPL	Conc. of TST	BPL			TST		
		RD	^1^DD	MC	RD	^1^DD	MC
(µg/mL)	(µg/mL)	%R	%R	%R	%R	%R	%R
4	32	100.18	100.12	100.01	99.95	99.84	99.24
3.5	28	99.62	100.56	100.01	99.78	100.52	99.91
3	24	100.01	100.45	100.09	100.48	100.35	99.76
2.5	20	100.22	100.94	100.62	101.19	99.87	99.25
2	16	100.52	100.71	100.71	99.96	100.61	99.98
Mean		100.11	100.56	100.29	100.27	100.24	99.63
±		±	±	±	±	±	±
RSD		0.330	0.305	0.346	0.573	0.359	0.359

### Application of the methods

Due to the unavailability of the dosage form in the Egyptian market, a laboratory-prepared tablet was prepared as described in "Application to laboratory-prepared tablets". The employed procedures were successful in quantifying BPL and TST in lab prepared tablets (Table 5), with minimal influence from excipients, as evidenced by the findings of the standard addition approach (Table 6).

**Table 6:** Application of the standard addition technique in qualitative analysis of BPL and TST by applying the proposed methods.

Pharmaceutical	BPL	BPL			TST	TST		
Taken	Added	RD	^1^DD	MC	Added	RD	^1^DD	MC
(µg/mL)	(µg/mL)	%R	%R	%R	(µg/mL)	%R	%R	%R
2 + 16	4	99.56	99.46	99.32	4	99.37	100.59	98.76
	8	100.53	99.65	100.47	8	98.49	99.47	99.27
	12	100.4	99.71	100.16	12	98.74	100.69	99.87
Mean		100.16	99.61	99.99		98.87	100.25	99.30
±		±	±	±		±	±	±
RSD		0.525	0.134	0.593		0.459	0.676	0.560

## Statistical analysis

Table 7 provides a statistical comparison of the findings achieved through the proposed and reported method, which was area under curve (AUC) by calculating AUC between 287 and 304 nm for the determination of telmisartan and 219–229 nm for the determination of bisoprolol fumarate^15^. The estimated t and F values were less than the theoretical ones, showing that there was no substantial difference in accuracy and precision between the proposed and reported procedures.

**Table 7:** Statistical comparison of the outcomes from the suggested approach with the reported method for the determination of BPL and TST.

Parameter	BPL			BPL reporter methods^9^	TST			TST reporter methods^9^
	RD	^1^DD	MC		RD	^1^DD	MC	
Mean	100.04	100.66	100.22	100.51	100.31	100.45	99.74	100.05
n	5	5	5	5	5	5	5	5
SD	0.469	0.505	0.398	0.774	0.543	0.542	0.650	0.660
RSD%	0.469	0.502	0.397	0.770	0.541	0.540	0.651	0.660
Variance	0.220	0.255	0.158	0.599	0.294	0.294	0.422	0.436
t-Stat (2.306)	1.160	0.106	0.752	–	0.665	1.053	0.757	–
F value (6.388)	2.719	2.347	3.789	–	1.482	1.484	1.034	–

The values in the parenthesis are the corresponding theoretical values of t and F at (P = 0.05).

## Greenness assessment

The level of greenness and sustainability of the suggested spectrophotometric approach was assessed in relation to three previously documented HPLC^1^, HP-TLC^2^, and spectrophotometry^9^ methods using the AGREE metric.The suggested method showed a far greater level of sustainability than the reported methods, according to analysis of the AGREE pictograms in Table 8. The use of water, a less harmful solvent than methanol, in the suggested approach explains this finding, since it replaces the latter with a safer alternative. Furthermore, when compared to HPLC and HPTLC, the spectrofluorometer was discovered to produce less waste and use less energy.

**Table 8:** Greenness comparison of the proposed method with the reported methods.

Item	Proposed method	Reported method^14^	Reported method^8^	Reported method^15^
Instrument	Spectrophotometer	HPLC	HP-TLC	Spectrophotometer
Solvent	Water	Amm. formate, methanol, and acetonitrile	Methanol, ethyl acetate, and acetic acid	Methanol
AGREE				

The Analytical GREEnness (AGREE) tool is a pictogram calculator that resembles a clock and contains 12 assessment criteria that match the 12 principles of green analytical chemistry. Every assessment criterion had a corresponding colour scale that went from red to green, and each grade ranged from 0 to 1. The pictogram's central portion showed the overall score and colour. The method received a green hue and an overall score of 0.84 on the AGREE assessment of the implemented approach (Table 8), showing that it is significantly green.

## Conclusion

Three recommended ratio manipulitaing spectrophotometric approaches were applied for precise and accurate quantification of the examined medications in their pure form or in pharmaceutical formulations.

Without the need for prior separation, even though the spectra of telmisartan and bisoprolol completely overlapped. The suggested approaches' exceptional simplicity, accuracy, and resilience, as well as their good greenness profile, which demonstrates a strong adherence of the applied approach to the principles of Green Analytical Chemistry by employing the AGREE greenness assessment metric, make them suitable for routine analysis and quality control.

## Data Availability

The datasets used and/or analyzed during the current study available from the corresponding author on reasonable request.
